# Therapeutic Bacteriophages for Gram-Negative Bacterial Infections in Animals and Humans

**DOI:** 10.20411/pai.v7i2.516

**Published:** 2022-10-17

**Authors:** Panagiotis Zagaliotis, Jordyn Michalik-Provasek, Jason J. Gill, Thomas J. Walsh

**Affiliations:** 1 Transplantation-Oncology Infectious Diseases Program, Weill Cornell Medicine New York, NY; 2 Center for Phage Technology, Texas A&M University, College Station, Texas; 3 Departments of Pediatrics and Microbiology & Immunology, Weill Cornell Medicine New York, NY; 4 Center for Innovative Therapeutics and Diagnostics, Richmond, VA; 5 Department of Pharmacology and Therapeutics, School of Pharmacy, Aristotle University of Thessaloniki, Greece

**Keywords:** bacteriophage, phage therapy, Gram-negative bacteria, antibacterial resistance, multi-drug resistance

## Abstract

Drug-resistant Gram-negative bacterial pathogens are an increasingly serious health threat causing worldwide nosocomial infections with high morbidity and mortality. Of these, the most prevalent and severe are *Pseudomonas aeruginosa, Klebsiella pneumoniae, Escherichia coli, Acinetobacter baumannii,* and *Salmonella typhimurium.* The extended use of antibiotics has led to the emergence of multidrug resistance in these bacteria. Drug-inactivating enzymes produced by these bacteria, as well as other resistance mechanisms, render drugs ineffective and make treatment of such infections more difficult and complicated. This makes the development of novel antimicrobial agents an urgent necessity. Bacteriophages, which are bacteria-killing viruses first discovered in 1915, have been used as therapeutic antimicrobials in the past, but their use was abandoned due to the widespread availability of antibiotics in the 20th century. The emergence, however, of drug-resistant pathogens has re-affirmed the need for bacteriophages as therapeutic strategies. This review describes the use of bacteriophages as novel agents to combat this rapidly emerging public health crisis by comprehensively enumerating and discussing the innovative use of bacteriophages in both animal models and in patients infected by Gram-negative bacteria.

## INTRODUCTION

Following the discovery of penicillin, the world of medicine entered “the golden era of antibiotics” [1]. Infections could be treated and kept under control by antimicrobial agents with unprecedented efficacy. However, the very success of antibiotics, as well as their broad availability, led to their widespread use and to the subsequent development of antimicrobial resistance by many pathogens [2]. Among the additional causes of antibiotic resistance are their inappropriate prescribing, extensive use in agriculture, and relatively small number of new antimicrobial agents with novel mechanisms of action [3].

The World Health Organization (WHO) has established a list of high priority pathogens on which research and development of new antimicrobial agents are imperative [3]. The 5 critical-priority pathogens are the Gram-negative bacteria *Acinetobacter baumannii, Pseudomonas aeruginosa,* and carbapenem-resistant *Enterobacteriaceae* (eg, *Klebsiella pneumoniae, Escherichia coli,* and *Enterobacter* spp.), while *Salmonella* spp. are considered of high priority [4].

### Challenges of multidrug-resistant Gram-negative bacterial infections

Gram-negative bacteria are particularly difficult to treat due to the presence of the outer cell membrane (containing lipopolysaccharide/endotoxin), which negatively affects permeability to antibiotics and is a major determinant of intrinsic antibiotic resistance [3]. Gram-negative rods are responsible for several types of infections, such as intraabdominal infections (IAIs), urinary tract infections (UTIs), and hospital-acquired pneumonia. More than 25% of all pathogen-related nosocomial infections in the United States are caused by these bacteria, with *P. aeruginosa, K. pneumoniae,* and *E. coli* infections constituting nearly 70% of all Gram-negative-related infections [5]. This renders Gram-negative infections an ongoing public health risk, which, in combination with antibiotic resistance, elevates the need for potential treatments for these infections.

In addition to the intrinsic antibiotic resistance due to their cell envelope structure, Gram-negative bacteria possess several other mechanisms of antibiotic resistance (Figure 1A). Among these mechanisms are limiting the uptake of a drug, modification of a drug target, inactivation of a drug, and active efflux of a drug [6].

**Figure 1A.**
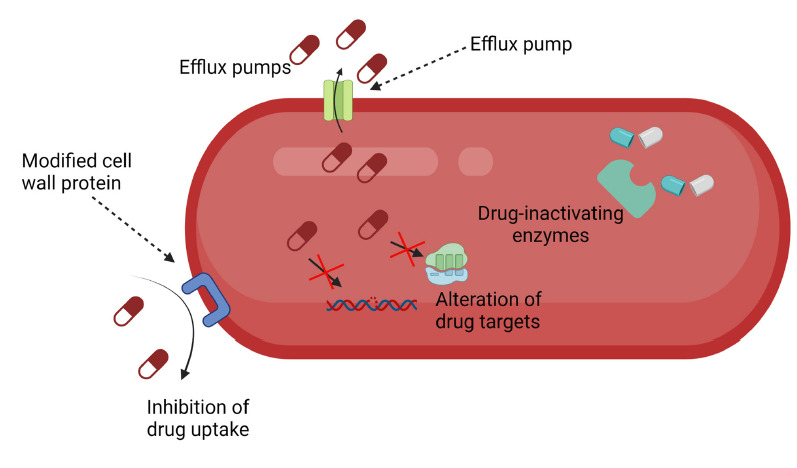
Mechanisms of bacterial resistance to antibiotics.

**Figure 1B.**
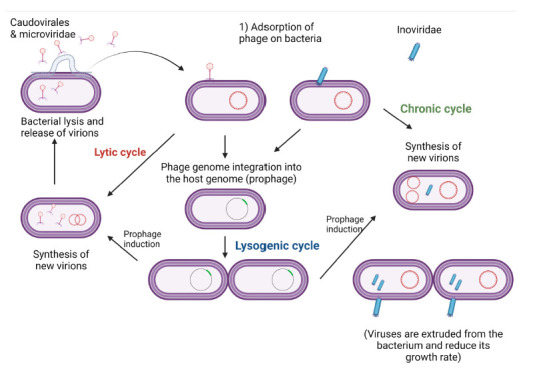
**Life cycles of phages.** Bacteriophages can either be lytic, in which case they cause lysis of the bacterial cell and release progeny within a short period of time, or lysogenic, in which case they inject their genome into the bacterial genome and stay dormant for a while until they get activated and release progeny. For therapeutic purposes, the preferred type of phage is lytic, as killing of the bacteria within a short period of time is necessary in treatment of bacterial infections.

The clinical utility of new antimicrobial agents designed to treat resistant bacteria is being eroded. For example, cefiderocol with its novel catechol-based iron transport mechanism for delivering a beta-lactam antimicrobial agent to the cell wall of multidrug-resistant Gram-negative bacteria may be threatened by a wide range of mechanisms of resistance [7]. Among these mechanisms are novel beta-lactamases, efflux pumps, porin mutations, and mutations of siderophore receptors, as well as modified penicillin-binding proteins. Heteroresistance among strains of *Acinetobacter baumannii* poses additional challenges to antimicrobial therapy by permitting a subpopulation of resistant organisms to emerge during the course of treatment and to transmit genetic information to the susceptible population [7].

Pathogens can be classified as multidrug resistant (MDR), extensively drug resistant (XDR), and pandrug resistant (PDR). While definitions for these types of resistance categories have varied, an initiative by the European Centre for Disease Prevention and Control (ECDC) and the Centers for Disease Control and Prevention (CDC) concluded with the following definitions: MDR was defined as acquired non-susceptibility to at least 1 agent in 3 or more antimicrobial categories, XDR was defined as non-susceptibility to at least 1 agent in all but 2 or fewer antimicrobial categories (ie, bacterial isolates remain susceptible to only 1 or 2 categories), and PDR was defined as non-susceptibility to all agents in all antimicrobial categories [8]. The bacteria examined in this review are either MDR or XDR, with some cases of PDR *A. baumannii, K. pneumoniae* and *P. aeruginosa* having also been reported [9].

Given the increasing resistance of bacteria to antibiotics and the relatively low number of newly available antibiotics, we have entered the “post-antibiotic era” [10], where bacterial infections are very difficult or sometimes impossible to treat with existing antibiotics. Therefore, the need for new treatment options is urgent. One such option is the use of bacteriophages, which are viruses that infect bacteria.

Bacteriophages were first discovered in 1915 by Frederick Twort, an English physician [11]. Two years later, the French-Canadian microbiologist Felix d' Herelle independently published similar observations, and would continue working with bacteriophages for most of his career [12]. Bacteriophages act by causing lysis of the bacterial cell with the recruitment of several mechanisms [13]. These mechanisms dictate the life cycle of the phages, which can be either lytic or lysogenic (or temperate)(Figure 1B). The mechanisms entail the use of holins to create pores in the cell membrane, as well as endolysins to degrade the peptidoglycan of the cell wall of Gram-negative and Gram-positive bacteria. These 2 types of enzymes are often sufficient to disrupt the bacterial cell envelope (cell membrane and cell wall) of Gram-positive bacteria. A third class of bacteriophage enzymes, spanins, is needed for lysis of Gram-negative bacteria. These enzymes fuse the inner and the outer cell membranes of Gram-negative bacteria, therefore causing lytic disruption [13].

In most cases, bacteriophages are administered to animals in the form of whole phage particles. Certain studies have also reported the use of bioengineered phage products and individual phage enzymes, such as endolysins, which are peptidoglycan-degrading enzymes that normally take part in the lysis of the bacterial cell wall [14–17].

Bacteriophages are relatively easy to isolate from the environment, with several methods having been reported [18]. Furthermore, they exhibit high host specificity, usually targeting only specific strains of bacteria and leaving the microbiota as well as eukaryotic cells unaffected. In addition, they have the advantage of self-replicating at the expense of their hosts, in contrast to conventional small molecule antibiotics.

As with many novel antibacterial treatment efforts however, the fact that many bacteriophages have shown *in vitro* activity does not automatically ensure their *in vivo* efficacy. Factors such as systemic clearance, pH, and route of administration increase the unpredictability of otherwise successful *in vitro* applications. Therefore, the objective of this article is to review the *in vivo* and clinical applications of phage therapy against Gram-negative bacterial infections, alone or in combination with antibiotics. Even though several review articles have been written with regard to phage therapy, they either discuss phage therapy as part of potential treatment alternatives against the so-called ESKAPE pathogens (*Enterococcus faecium, Staphylococcus aureus, Klebsiella pneumoniae, Acinetobacter baumannii, Pseudomonas aeruginosa*, and *Enterobacter* spp) [19], or they do not discuss extensively the aspects of phage kinetics, potential immune reactions, and histology outcomes of phage therapy [20]. This review further discusses the advantages, potential limitations, and potential developments that will optimize the future applications of phage therapy.

### Animal models of phage therapy

The use of bacteriophages has been reported in many studies of different animal models, mimicking the infections that can occur in humans. Translatable models of mice and rat infections have been used more widely [15, 16, 21–46], with infection models in rabbits, swine, sheep, or cattle having been used less frequently [47–51].

Most studies focus on the therapeutic efficacy of phage, but many also address phage kinetics, ie, the rate of clearance from the bloodstream and tissues [21–23, 26, 29, 52–55]. As phages have the ability to self-replicate and can be cleared at different rates according to conditions found in the tissues, these important pharmacokinetic aspects of phage therapy differ greatly from those of conventional antimicrobial agents. The route of administration also impacts the pharmacokinetics of bacteriophage *in vivo*. Gastric pH or the need to cross the gastrointestinal epithelium may limit the utility of oral administration of bacteriophage for systemic therapy. Other studies also examine the effect of phage on the infected tissues, as well as on the immune response of the host, by measuring pro-inflammatory cytokines, such as TNF-α and IL-6 [21–23, 26–28, 40, 42, 45, 46, 53, 55–59]. Measurements of these pro-inflammatory cytokines may serve as surrogate biomarkers for Gram-negative sepsis. The temporal patterns of serum pro-inflammatory cytokines generally parallel improvement in survival and reduction of residual bacterial tissue burden in animal models.

In studies of potential synergy between phage and host immune response, both immunocompe-tent and immunosuppressed animals were used to compare the differences in outcome [29].

### Pneumonia

Gram-negative bacilli are important causes of hospital-acquired pneumonia and chronic lung infections worldwide, with many animal models for treatment of Gram-negative bacillary pneumonia having been developed [21, 22]. Table 1 summarizes the *in vivo* studies of therapeutic bacteriophages against Gram-negative bacterial pneumonias.

**Table 1.** Animal Pneumonia Studies

Reference	Pathogen	Animal Model	Dose & Route of Administration	Outcome	Phage Kinetics	Histology & Cytokines
[56]	*A. baumannii*	Mouse pneumonia	Single phage dose, IN	>2 log_10_ reduction in CFU by day 1, bacteria were cleared from lungs by day 3. Bacteremic group: Died by day 5. Treated groups survived as follows: a) MOI = 10, 100%, b) MOI = 1, 60%, c) MOI = 0.1, 30%	Treatment group (lungs): Day 1 – 10^12.2^ log_10_ PFU, Day 3 – 10^7.7^ log_10_ PFU Phage-only group: Day 1 – 10^6.2^ log_10_ PFU, Day 3 – 10^2.3^ log_10_ PFU	Untreated group: Severe alveolar wall thickening, hemorrhaging in alveolar space. Treated group: Only mild to moderate thickening of the alveolar walls. TNF-α and IL-6 levels significantly reduced in treated group
[23]	*A. baumannii*	Mouse pneumonia	3 × 10^5^ / 3 × 10^6^ / 3 × 10^7^ PFU/mouse, IN	3 log_10_ reduction for treated group, 24 hpi. P<0.0001 MOI = 10 increased survival by 90%. Furthermore, mice showed fewer clinical signs.	Phage-only group (no bacteria): 4.22 × 10^6^ PFU/lung Phage-treated group (bacterial infection): 8.3 × 10^7^ PFU/lung. 1 log difference at 24 hpi.	Non-treated group: severe thickening of alveolar wall, severe cell infiltration of perivascular and peribronchial region. Phage-treated group: no inflammation, milder thickening of alveolar wall
[52]	*A. baumannii*	Mouse pneumonia	10^8^ PFU/mouse IN, Q=24h	2 log_10_ reduction in CFU between treated and non-treated group on days 3 and 4. Treated group – 35% survival, non-treated group – 15% survival 7 dpi	N/A	Immune responses to phage-only: 20% increase in serum IgE, slight increases in serum GM-CSF, IL-2, IL-10, and IL-17a, after IP, IN, and oral administration
[53]	*A. baumannii*	Mouse pneumonia	2 × 10^8^ PFU/mouse, IN	4 log_10_ reduction in CFU in the treated group, compared with the non-treated one, 3 dpi. Treated group: 100% survival at 6 dpi. Untreated group: 0% survival at 6 dpi.	Phage-only group: 2 log_10_ less PFU than the infected group in each time point (ie,12h, 24h, etc.)	Non-treated mice: heavier rates of congestion and edema than phage-treated mice
[24]	*E. coli*	Mouse pneumonia	5 × 10^9^ PFU/mouse, IN or IP, 5 × 10^10^ PFU/mouse, IP	3 log_10_ reduction in CFU/g with both routes	Phage counts were the same in 2 groups 17h post infection, even though IP dose was initially 10 times higher	N/A
[60]	*E. coli*	Mouse pneumonia	MOIs 0.3, 3, or 10 (1.2 × 10^7^, 1.2 × 10^8^ or 4 × 10^9^ PFU/mouse), IN	100% survival in treated mice on day 3, compared with 0% in control group on day 3, at all MOIs. 2 log_10_ reduction in bacterial burden 6-16 hpi Adaptation of the phage to the VABP strain increased survival from 20% to 75%	1.1 log_10_ increase in PFU counts from the 6h to the 16h mark after infection	PMN engulfment in BAL fluid and chemokine significantly lower in phage-treated group, 16h post infection
[25]	*P. aeruginosa*	Mouse pneumonia	900μg/mouse, IN or IT	20% survival for IN administration, 70% survival for IT administration (10-day period)	N/A	N/A
[61]	*P. aeruginosa*	Mouse pneumonia	10^6^ or 2 × 10^7^ PFU/mouse, IN	100% survival with PhiKZ phage at MOI = 20, 0% at MOI = 0.1 90% survival with PAK_P1 at MOI = 0.1	N/A	N/A
[26]	*P. aeruginosa*	Mouse pneumonia	2-5 × 10^9^ PFU/ mouse, IN	3 log_10_ decrease in CFU in treated mice	N/A	N/A
[27]	*P. aeruginosa*	Mouse pneumonia	2 × 10^7^ PFU/mouse, IN, 2 doses at 24 and 36h post infection OR at 48 and 60h post infection OR at 6 days post infection	3 log_10_ reduction in CFU/g in lungs of treated mice, with complete bacterial clearance eventually, in the first 2 groups Complete clearance in 70% of mice and 1 log_10_ reduction in the remaining 30% in treated mice 6 days post infection, in third group	N/A	N/A
[47]	*K. pneumoniae*	Mouse pneumonia	**Therapeutic use:** Phage only: 10^4^ PFU/mouse, IP, 6h, 24h, and 72h post infection Liposome entrapped phage: 10^2^ PFU/mouse, IP, 6h, 24h, 48h, and 72h post infection **Prophylactic use:** Phage only: 10^4^ PFU/mouse, IP, 1h, 3h, 6h, and 24h prior to infection Liposome entrapped phage: 10^2^ PFU/mouse, IP, 6h, 24h, 48h, and 72h prior to infection	**Therapeutic use:** Phage only group: 6h dose cleared all organisms from lungs. 24h dose reduced CFU/g by 2.8 log_10_ 48h dose did not show any significant reduction Liposome entrapped phage group: 6h and 24h doses cleared all bacteria. 48h and 72h doses reduced bacteria numbers by 5.2 and 2.2 log_10_, respectively, with complete clearance 72 hours after administration of the phage **Prophylactic use:** Phage only group: 3h and 6h doses prior to infection completely inhibited infection. 24h prior – no difference with control group Liposome entrapped phage group: 6h, 24h, and 48h doses before infection, completely inhibited infection. 72h dose did not have any effect	Non-infected mice which were given phage had higher retention than infected ones for up to 3-4 days	Phage-treated group: IL-1β and TNF-α reduced in phage-treated group, either 6h prior to infection or after infection. IL-10 was increased in treated group. No difference when administered 48h after infection. Liposome-entrapped phage group: Cytokine levels displayed more significant difference compared with both control group and phage-only group. Doses at all times lead to the differences, except for dose 72h prior to infection. Both prophylactic and therapeutic dose protected lungs from neutrophil infiltration and damage.
[54]	*K. pneumoniae*	Mouse pneumonia	10^9^ (MOI=10) or 10^10^ (MOI=100) PFU/mouse, IP	MOI = 10 resulted in 5 log_10_ reduction in CFU 3 days post infection MOI = 100 resulted in 7 log_10_ reduction	N/A	No lung enlargement or abscesses in treated groups
[28]	*P. aeruginosa*	Mink pneumonia	10^8^, 10^9^, and 10^10^ PFU/mink, by ultrasonic nebulization	10^10^ PFU reduced CFU by 1.5 log_10_ and 80% survival 10^9^ PFU reduced CFU by 1 log_10_ and 20% survival 10^8^ PFU reduced CFU by 0.5 log_10_ and 0% survival	N/A	N/A
[29]	*P. aeruginosa*	Mouse pneumonia	10^8^ PFU/mouse, inh, at 2h after infection	100% survival in treated mice compared with 0% in untreated ones, as well as complete clearance of bacteria 48h after treatment. Intact innate immunity was required for phage treatment success. Neutropenic mice could not be saved even with 100-fold less bacterial inoculum	Phage was cleared from mice at a rate of 0.5 log_10_/day, which resulted in efficacy of treatment up to 4 days after phage administration (MOI = 0.1 at that point)	N/A
[55]	*P. aeruginosa*	Mouse pneumonia	10^7^ PFU/mouse (MOI = 10), IP	Phage-treated groups: 66% and 83% survival on day 12, compared with 0% survival (all died by day 3) in untreated group 1–2 log_10_ reduction in CFU/g in lungs with both phages on day 1, 6 log_10_ reduction from both phages on day 5, with some mice undergoing complete clearance of bacteria on day 5	N/A	Untreated group: severe alveolar wall thickening and neutrophil infiltration. Treated group: Mild or no histological findings. TNF-a and IL-6 levels equal on day 1, but significantly reduced on day 5 in treated group
[62]	*P. aeruginosa*	Mouse pneumonia	10^7^ PFU/mouse, inh, single dose at 2h post infection	5 log_10_ reduction in CFU/g in lungs at 24h	Phage steadily increased in the first 24 hours, reaching 1–2 log_10_ higher concentrations at 24h after administration in the lungs and plasma	N/A
[30]	*E. coli*	Chicken pneumonia	Approx. 10^9^ PFU/mL, aerosolized, or IM, 2h, 24h, or 48h after bacterial inoculation	Aerosol administration directly after *E. coli* infection increased survival from 50% to 80%. IM injection increased survival as follows: from 47% to 83% when given on day 0, from 54% to 90% when given on day 1 from 56% to 80%% when given on day 2	Aerosol: a few birds had an average of 10^2^ PFU/mL1h after administration, no phage at 24h and 48h. IM injection: Birds had a 10^4^ PFU/mL in blood 6h after administration, and approximately 70 PFU/mL 24h after	Non-treated birds: colibacillosis lesions, pericarditis and increased spleen and liver weights

Abbreviations: CFU: colony forming unit; PFU: plaque forming unit; MOI: multiplicity of infection; IN: intranasal; IT: intratracheal; dpi: days post-inoculation; N/A: not applicable

In most cases, intranasal inoculation is the preferred route of administration [13–27, 52, 53, 56, 60, 61]. This route ensures that the phage would reach the bacteria in the lungs in high concentrations and achieve a faster therapeutic outcome. Some models also use the inhalational or intraperitoneal routes [28–30, 47, 54, 55, 62]. Independent of the route of administration, most phage doses range from 10^7^ plaque-forming units (PFU) to 10^10^ PFU, and all models include at least 1 dose that is 10-fold higher than that of the bacterial burden at the time of administration. This ratio allows for a multiplicity of infection (MOI) of 10. Singla et al [47] reported the only study that used a lower dose (10^4^ PFU), as it aimed to examine the effect of liposomal entrapment of the phage on the therapeutic outcome. The phage dose for the liposome-entrapped phage in that study was as low as 10^2^ PFU.

The outcome of treatment in these studies was determined by both the dose and route of administration, as well as the timing of the dosing. Both survival and reduction of colony-forming units (CFU) were dose-dependent and were improved at MOIs ≥ 10, while a better outcome was achieved for quicker initiation in therapeutic models.

All studies that examined bacteriophage kinetics showed that phage titers were higher at the same timepoints in bacteria-infected groups than in phage-only groups (animals free from bacterial infection), both in tissue and in blood. This indicates that phage population expands when encountering and replicating in host bacteria, leading to higher *in vivo* concentrations in infected animals.

Histopathological examination of the tissues in some studies revealed that phage treatment may also protect lungs of treated animals from alveolar wall thickening and neutrophil infiltration, compared to untreated controls [23, 53–56, 60].

### Bacteremia

The presence of bacteria in the bloodstream (bacteremia) can cause sepsis and septic shock, especially when a patient is immunocompromised [31]. Animal models of bacteremia have used phages to treat this potentially fatal condition (Table 2).

**Table 2.** Animal Bacteremia Studies

Reference	Pathogen	Animal Model	Dose & Route of Administration	Outcome	Phage Kinetics	Histology & Cytokines
[14]	*A. baumannii*	Mouse bacteremia	10^8^ PFU/ml, IP	Treated group: 100% survival at 6 weeks Untreated group: 0% survival	N/A	N/A
[15]	*K. pneumoniae*	Mouse bacteremia	Prophylactic treatment: Depolymerase 50μg/mouse, IP, single dose 6h prior to infection. Therapeutic treatment: Depolymerase 50μg/mouse, IP, single dose 30 min after infection	100% survival in mice treated with Dp42 in both prophylactic and therapeutic treatment. When infected with Dp42 pretreated *K. pneumoniae*, mice showed 80% survival compared with 0% of untreated mice. CFU/g in liver and lungs were 3 log_10_ lower than in untreated group, and 5 log_10_ lower in spleen	N/A	N/A
[17]	*E. coli*	Rat bacteremia	0.25 μg of depolymerase, IP single dose	Untreated group: 0-20% survival. Treated group, when EndoE was given within 24h: 90-100% survival. Treatment was less effective when starting on day 3	N/A	N/A
[24]	*E. coli*	Mouse bacteremia	6 × 10^10^ PFU/mouse IP, single or double dose	Single or double dose was not able to prevent the death of animals, but resulted in a ~1.5 log_10_ reduction in CFU/gr	Phage counts were 1 log_10_ higher in double dose group than in single dose group	N/A
[30]	*K. pneumoniae*	Mouse bacteremia	1.75 × 10^8^ PFU/mouse, IP, single dose	Untreated group: 0% survival. Phage-treated group: 100% survival when phage was administered 10 min after bacterial challenge. 12.5% survival when administered 1h after bacterial challenge. 0% survival when administered 3h after bacterial challenge	N/A	N/A
[32]	*A. baumannii*	Mouse bacteremia	5 × 10^8^ PFU, IP, Q=24h for 6 days	Phage-treated group: 100% survival. Untreated group: 10% survival. No bacteria in kidneys and liver in treated group. High bacterial burden (10^9^ CFU/gr) in untreated group	N/A	N/A
[65]	*E. coli*	Mouse bacteremia	From 10^3^ to 6 × 10^9^ PFU/mouse, IP, single dose	With a MOI from 10^−4^ to 200, 100% survival rate. Lower MOIs (10^−5^ and lower) decreased survival to 0-20%.	10^9^ PFU/mL were retained up to 24h after injection, 10^6^ PFU/mL 48h after	N/A
[33]	*E. coli*	Mouse bacteremia	10^8^ PFU/mouse, IV, single dose at 10 min, 1h, or 2h after infection	95-100% survival if administered within 1h, 33% survival if administered within 2 hours. No untreated mice survived.	Phage was detected up to 2 weeks after infection and administration. 3 × 10^4^ PFU/mL in the spleen and 2 × 10^3^ PFU/mL in the brain	N/A
[35]	*E. coli, K. pneumoniae* and *P. aeruginosa*	Mouse bacteremia	10^9^ PFU/mouse, IP at 5h, 14h, and 18h after infection	100% survival in all pathogen-infected mice after 5 days, compared with 0% in control. Single phage only rescued 60% of mice. In *E. coli* and *K. pneumoniae* co-infection, different phage cocktails showed different survival rates, 100%, 40%, and 0% for each cocktail	N/A	N/A
[64]	*P. aeruginosa*	Mouse bacteremia	5 × 10^6^, 5 × 10^7^, or 5 × 10^7^ PFU/mouse, IP, single dose	Immunocompetent mice: 100% survival with MOIs of 10 and 100 (5 × 10^7^ and 5 × 10^8^ PFU); 80% survival with MOI = 1. MOIs of 10 and 100 completely cleared bacteria from blood and lungs within 48h Neutropenic mice: Phage could only prolong survival, but none survived eventually	Phage was cleared from all organs in both infected and non-infected mice within 72h. In infected mice, phage persisted in the spleen for up to 96h	N/A
[36]	*P. aeruginosa*	Mouse bacteremia	10^9^ PFU/mouse, IP, single dose	Phage-treated group: 92% survival Untreated group: 7.4% survival.	Phage titers increased in blood, liver, and spleen during the first 12h and then decreased. Cleared from blood within 24h and from both blood and liver at 48h	N/A
[63]	*P. aeruginosa*	Mouse peritonitis-sepsis	2 × 10^8^ PFU/mouse, IM or IP, single dose at 6h post infection (2 different phages were administered)	Survival with both phages was 100% or 80%, compared with 0% in untreated group 2-3 log_10_ reduction of CFU/g in blood, liver, and spleen with one phage, 0.5 - 1 log_10_ reduction with the other	Phage were detectable in blood, liver, and lung up to 36h post infection. They were cleared faster from blood and lung compared with liver. IM administered phage persisted longer in the organs. Also, one type had higher PFU/g at all timepoints compared with the other	N/A
[37]	*P. aeruginosa*	Mouse bacteremia	10^4^ PFU/mouse, IV, single dose at 1h after infection	8 log_10_ reduction in CFU after 2.5h, compared with non-treated mice. Lower concentrations than 10^4^ were ineffective in treatment of the infection.	Phages remained in the liver and spleen for 36h in healthy mice, but were minimally detectable in urine.	N/A
[38]	*P. aeruginosa*	Mouse bacteremia	Lysocin 2.5, 5, 12.5, and 25 mg/kg, IP, 3h post infection	73%, 80%, 93%, and 100% survival with each dose respectively, compared with 37% survival of control group	N/A	N/A
[39]	*A. baumannii*	Mouse bacteremia	1mg of lysin, transcatheter, 2 doses, 4 hours apart	2log_10_ reduction in catheter-mimicking model, with phage administration 24 hpi in bacteremic mice. Phage-treated group: 50% survival. Untreated group: 10% survival	N/A	N/A

Abbreviations: IP: intraperitoneal; PFU: plaque forming unit; N/A: not applicable; IM: intramuscular; IV: intravenous; CFU: colony forming unit

The most common route of administration in such studies is intraperitoneal or intravenous injection [16, 24, 32–37, 63–65]. Four studies also examined the use of isolated phage peptides, rather than whole phage particles, for the treatment of bacteremia in mice and rats [17, 19, 38, 39]. In all these studies, intraperitoneal injection is the preferred route of administration. All phage doses aimed for MOIs ≥ 1, while Wang et al [65] explored the efficacy of phage treatment at MOIs ranging from 0.0001 to 200.

As with pneumonia, the result of phage treatment was both dose-dependent and time-dependent, with the time of initiation of the treatment being more important in rescuing the animals. Higher survival rate was inversely related to time of initiation of phage therapy. This effect was observed with either the use of peptides (such as lysins) or whole phage particles in cocktails. Notably, no dose of bacteriophage was sufficient to rescue animals if treatment started too late, as shown by Horvath et al [34], while similar observations were made in the case of depolymerase (lysin) administration [19]. The immune system of the animals is another important aspect which plays a role in treatment outcome. As previously stated [64], immunocompetent mice achieved high survival rates compared with neutropenic mice, none of which survived in the study of bacteremia caused by *P. aeruginosa*. As with the other studies, the result of treatment was also dose-dependent, with MOIs of 10 and 100 showing 100% survival, compared with 80% survival with MOI of 1. The results of phage therapy in different laboratory animal models are a function of inoculum size, level of immunosuppression, genetics of the animal, timing of initiation of therapy, and dosage schedule of the intervention. When such conditions are optimized, phage therapy and antibacterial therapy may be highly effective in treating the target infectious disease.

For kinetics, the existence of bacteria in the bloodstream was important for the retention of phage, with non-infected animals clearing phage faster. Phage also persisted longer in the spleen and secondarily in the liver, compared with other organs or the blood [33, 36, 37].

### Wound infections

Infections of open wounds by Gram-negative bacilli present an urgent necessity for treatment, as they can cause locally progressive tissue injury and bacteremia. Burns, trauma, and pressure injuries are especially challenging infections in hospitalized patients. Most animal models that address these infections in the preclinical setting involve the use of mice [37–39, 55, 63, 66, 67], with one study using chickens as well as mice [41] (Table 3).

**Table 3.** Animal Wound Infection Studies

Reference	Pathogen	Animal Model	Dose & Route of Administration	Outcome	Phage Kinetics	Histology & Cytokines
[25]	*P. aeruginosa*	Mouse wound infection	Lysin, 100, 200, or 300 μg/mouse, topically, single dose	100 μg – 1 log_10_ reduction in CFU 300μg – >2 log_10_ reduction in CFU	N/A	N/A
[66]	*A. baumannii*	Mouse wound infection	5 × 10^8^ PFU, SC, or by direct pipetting on the wound	Wounds in local application of phage were significantly smaller than non-treated group and systemic application (differences between systemic-application group and non-treated group were minimal)	N/A	N/A
[67]	*A. baumannii*	Mouse wound infection	10^8^ PFU/mouse, IP, and 5 × 10^7^ PFU/mouse topically in Tegaderm dressing, at 4h, 24h, and 48h post infection	Non-treated mice did not see reduction or closure of wound. Prophylactic treatment and post-infection treatment group did not show great differences. The 3rd group, non-prophylactic treatment and phage post infection, showed the highest reduction in wound size and the wound had closed by day 17	24h following 10^8^ PFU/mouse IP administration, the spleen had 10^5^ PFU/g, the liver had 10^3^ PFU/g and the lymph nodes had from 10^3^ - 5 × 10^7^ PFU/gr. Control group had no detectable phage in any of these organs	N/A
[40]	*E. coli*	Mouse wound infection	10^9^ PFU, topically	No difference in CFU counts was observed in MG1655 strain. The phagebody (PB10) completely cleared all bacteria from the wound (ΔwaaG strains) in comparison with control group and T3 phage-treated group)	N/A	N/A
[58]	*E. coli*	Mouse wound infection	10^10^ PFU/mouse, single dose, topically	A single dose of phage had the same efficacy as repeated doses of antibiotics and had cleared bacteria from the wound by day 3	N/A	N/A
[41]	*K. pneumoniae*	Mouse wound infection	5 × 10^6^ PFU/mouse, topically. 7 groups of mice: One control, 5 with single phage and one with a cocktail of phages	Untreated group CFU/g: 10^8.8^ Group 2: 10^4.3^ CFU/g, Group 3: 10^4.6^ CFU/g, Group 4: 10^4.4^CFU/g, Group 5: 10^4.1^ CFU/g, Group 6: 10^4.2^ CFU/g. Group 7 (cocktail): 10^3^ CFU/g Wound contraction and re-epithelization of the wound was fastest in the cocktail group, and slowest in the untreated group	Similar pattern for all groups: Day 1: 10^5^ – 10^6^ PFU, Day 3: 10^6^ PFU, Day 5: 10^4^ – 10^5^ PFU, Day 7: 10^2^ – 10^4^ PFU, Day 10: 10^2^ PFU	Untreated group: complete destruction of superficial skin layers along with partial fusion of collagen and dense inflammation. Significant increase in neutrophil infiltration and profound inflammation was observed in the skin. Phage-treated groups: healing in the form of collagen and fibroblastic proliferation and regeneration of epidermis with mild infiltration of lymphocytes.
[57]	*K. pneumoniae*	Mouse wound infection	Group 1: Untreated control Group 2: Phage 10^5^ PFU, IP Group 3: Liposome entrapped phage 10^5^ PFU, IP Group 4: Liposome alone, IP, all single dose	Bacterial counts kept increasing in untreated and liposome-only (control) groups for up to 72h, in blood, skin, and liver. Phage-treated and liposome-phage groups showed continuous reduction at 24h, 48h, and 72h, between 4 and 6 log_10_ reduction, with liposome entrapped phage group showing about 1 log_10_ lower counts than phage-only group at all timepoints	Phage alone remained in liver, spleen, and blood up to 36h after injection(peak at 6h) Liposome entrapped phage persisted in these organs up to 72h (peak at 6h) and up to 120h in the spleen (10^2.5^ PFU/gr)	Untreated animals (control) showed loss of epidermis and massive infiltration of neutrophils extending up to the muscle layer. Loss of distinction among individual collagen fibers. Thermally injured and infected mice, receiving CP showed regeneration of epidermis along with mild infiltration of neutrophils. Maximum healing was observed in mice administered with LCP
[68]	*K. pneumoniae*	Mouse wound infection	5 × 10^9^ PFU/mouse (MOI = 200), topically, single dose	63.33% survival in treated group at 7 days compared with 0% in control group	N/A	
[69]	*P. aeruginosa*	Mouse wound infection	Topically via nanofibrous dressing, single dose (dose not mentioned)	Wound size started decreasing on day 1 with all agents used, including antibiotics and phages, and the wound was completely healed on day 12 from either one	N/A	Increased collagen deposition and faster dermis formation on the site of the wound in the phage-treated group, compared with the untreated one

Abbreviations: IP: intraperitoneal; PFU: plaque forming unit; N/A: not applicable; IM: intramuscular; IV: intravenous; CFU: colony forming unit; SC: subcutaneous

Wound dressings are used to apply bacteriophages for topical administration in these infections. Two models, one of which examined the effect of liposome encapsulation on the efficacy of the phage, used intraperitoneal injections [37, 67]. All doses fell within 5x10^6^ – 10^10^ PFU for whole phage particles. Chadha et al [57], who examined the effect of liposome encapsulation of the phage, used 10^5^ PFU for both phage-only and liposome-entrapped phage cocktails, while Raz et al [25], who used a phage lysin, administered 100-300 μg/mouse topically.

In all models, a complete healing or significant reduction in the size of the wound was observed, with bacterial colony forming units (CFU) on the wound reduced 10-fold to 10^5^-fold. Liposome entrapment also increased the efficacy of treatment, leading to a greater reduction in CFU compared with the phage-only group [67]. The use of a phage cocktail composed of 2 or more bacteriophages was also shown to have a better impact on the outcome of the treatment when compared with single phage administration [66].

Three of the studies included an analysis of phage kinetics, 1 for topical administration [66] and the other 2 for intraperitoneal (IP) injection [37, 67]. In IP administration, the spleen was the organ with longest phage persistence, followed by the liver, while the entrapment of phages in liposomes reduced their clearance, maintaining them longer in the spleen, liver, and other tissues.

In those studies that histologically examined wounds [41, 58, 67], phage administration reduced the extent of inflammation and superficial skin destruction, as well as neutrophil infiltration, while liposome entrapment enhanced this activity.

### Other infections

Gram-negative bacilli are responsible for other infections, such as those involving the gastrointestinal (GI) tract, brain, cornea, the urinary tract, and muscle. Models of bacteriophage therapy for these conditions have been investigated and are reported in Table 4.

**Table 4.** Other Animal Infection Studies

Reference	Pathogen	Animal Model	Dose & Route of Administration	Outcome	Phage Kinetics	Histology & Cytokines
[16]	*E. coli*	Mouse thigh infection	Three different types of depolymerase, 0.1, 0.4, or 1 mg/kg, IM	10% survival for non-treated groups. K1 depolymerase: 95% survival K5: 100% survival K30: 70% survival	N/A	Clinical evaluation showed no signs of health decline, weight loss etc
[24]	*E. coli*	Mouse UTI	10^10^ PFU/mouse, IP	2 log_10_ reduction in CFU/g in kidneys	10^9^ PFU/g in the kidneys, 48h post infection	N/A
[63]	*P. aeruginosa*	Mouse peritonitis	2 × 10^8^ PFU/mouse, IM or IP, single dose at 6h post infection (2 different phages were administered)	Survival with both phages was 100% or 80% with D3112 phage compared with 0% in untreated group or with MP22 phage. 2-3 log_10_ reduction of CFU/g in blood, liver, and spleen with one phage, 0.5 - 1 log_10_ reduction with the other	Phage were detectable in blood, liver, and lung up to 36h post infection. They were cleared faster from blood and lung compared with liver. IM administered phage persisted longer in the organs. Also, one type had higher PFU/g at all timepoints compared with the other	N/A
[42]	*E. coli*	Mouse UTI	3 × 10^11^ PFU/mouse (MOI = 60), IP	90% survival of phage-treated group compared with 0% of control, 3 dpi	Phage particles were detected in liver, kidney, spleen, and lungs 4h post infection, and remained relatively stable up to 24h post infection in these organs.	N/A
[43]	*E. coli*	Rat GI infection	7.2 × 10^7^ PFU/rat, TD or orally	0% survival in untreated group - 83.3% survival in treated group	Infection-free rats: Transdermal administration: 1h after admin, phage titer in serum was high and remained elevated 24h after injection. Oral administration: No phage was detected in serum Infected rats: Phage started multiplying at 2h and peaked at 24 h (105 PFU). Phage was mostly found in the intestine, as there were hosts to infect, but not in the stomach, even though phage were orally administered	Phage-treated group showed no damage or lesions. Untreated group: dense lymph-omononuclear infiltration with oedema in lamina propria and inflammation of Peyerâ€™s patch. No major alterations in kidneys, liver, and stomach. IL-6 was significantly reduced in phage-treated group
[75]	*P. aeruginosa*	Mouse keratitis	Topically (eye drops), single dose	Mild or no corneal opacities in treated mice, in comparison with severe corneal perforation in untreated ones	N/A	Untreated mice: Destroyed stromal structure of cornea, severe neutrophil infiltration, high numbers of *P. aeruginosa* in the abscesses. Treated mice: Normal cornea, few detectable bacteria.
[70]	*E. coli*	Mouse thigh infection	K1 dep (dependent): 100 PFU/mouse, IM K1 ind (independent): 10^8^ PFU/mouse, IM	K1 dep saved 94% of mice at 100 PFU. In contrast, K1-ind phage at 10^8^ PFU were not enough to save mice (19% survival)	K1-ind phages: 3 hpi: 10^8^ PFU 6 hpi: 10^9^ PFU K1-dep phages: 3hpi: 10^9^PFU 6 hpi: 10^10^ PFU	N/A
[45]	*E. coli*	Mouse intramuscular and intracerebral infection	of 3 × 10^8^ PFU/mouse, IM or IV	Intramuscular infection survival: non-treated group 7%, phage-treated group 93% 3 log_10_ reduction in CFU/g in 30 min, 3 log_10_ reduction in blood and 5 log_10_ reduction in liver. Intracerebral infection Survival: non-treated group 5%. Phage-treated group: 60% 5 log_10_ reduction in CFU/g in brain in 120 min	Intramuscular infection non-infected mice had a 3 log_10_ reduction in phage in the muscle and spleen within 30 min, compared with bacteria-in-fected group.	N/A
[73]	*E. coli*	Rabbit GI colonization	10^9^ PFU/rabbit via stomach tube	Non-treated rabbits started dying on day 4. At the end of the experiment, 90% of animals had died. Treated animals did not evade death, but their survival was postponed until day 7.	After administration of phage only, all the organs had 10^3^-10^5^ PFU/g phage until day 4. After day 7, phage in all organs was <10 PFU/g, except for the spleen, in which it remained around 10^3^ PFU/gr. No phage was detected in plasma on any of the days In bacteria-in-oculated groups, phage in feces was approx. 10^9^ PFU/gr	N/A
[74]	*E. coli*	Pig diarrhea	5 trials: Trial 1: 10^10^ PFU/pig, orally Trial 2: 10^10^ PFU/pig, orally, but pigs were treated with antacids prior to challenge Trial 3: Prophylactic treatment, 15 min after bacterial inoculation Trials 4-5: Therapeutic use. Phage was administered 24h after bacterial inoculation	Pigs that received phage mixture showed reduced duration of diarrhea and score of diarrhea (severity) in all trials. In trials 4 and 5, after therapeutic application of phage, CFU/g was reduced by 1-1.5 log_10_ in treated group, in comparison with the untreated group	N/A	N/A
[76]	*E. coli*	Mouse peritonitis	10^11^ PFU/mouse (one group with LyD - lysis deficient phage, the other with WT phage), IP	81% survival in LyD-treated group, 55% in WT-treated, 33% in latamoxef-treat-ed, no mice survived in control group 7 log_10_ reduction in CFU/g in all treated groups in 6 hours, 8 log_10_ reduction in 12 hours	N/A	TNF-α and IL-6 levels were not different at 6h, but LyD group had significantly lower TNF-α levels than all groups at 12h. IL-6 levels at 12h were lower than control group for all treated groups
[77]	*K. pneumoniae*	Mouse liver abscesses	2 × 10^5^, 2 × 10^6^, 2 × 10^7^ or 2 × 10^8^ PFU/mouse, oral or IP	Untreated group: 10% survival IP treated group: 2 × 10^5^ PFU - 30% survival 2 × 10^6^ PFU - 90% CFU/g was not detected in liver or blood of treated mice at 6h, 24h, and 72h post infection	N/A	AST and ALT levels were lower in treated groups compared with untreated groups. TNF-α, IFN-γ, MCP-1, IL-10, and IL-6 were all at the same level as naive mice, compared with untreated group which had very elevated cytokine levels
[78]	*E. coli*	Sheep GI infection	10^11^ PFU/sheep, orally, single dose	2 log_10_ reduction of CFU in caecum and rectum, 2 days after start of treatment	N/A	N/A
[79]	*E. coli*	Rabbit ileal loop	10^6^ PFU/rab-bit, orally	Fluid accumulation in ileal loop was significantly inhibited in phage-treated group compared with untreated control. A 7 log_10_ reduction in CFU/g was also observed	N/A	N/A
[80]	*E. coli*	Rat GI colonization	Phage, 10^7^ PFU/rat, in drinking water. 10^7^ PFU/rat, orally, 3 times a day. 10^6^ PFU/rat, in vegetable capsules, 3 times a day, Q = 2h for 20 days Rats were separated in 2 trials: One only included the naturally inhabiting *E.coli* strains of rats, the other included inoculation with 60 human pathogenic strains	**1st trial (rat *E. coli***): For drinking water phage: 2.3 log_10_ reduction in CFU/g on day 7, gradual increase in bacterial load on day 14, same as control group on day 20 For oral gavage phage: 1.8 log_10_ reduction, starting on day 2 to day 12. On day 12, CFU started to rise again and reached control group on day 20 Vegetable capsules: 3 log_10_ reduction on day 6. Started to rise again on day 12 and remained 1 log_10_ lower on day 20 **2nd trial (human pathogenic *E. coli* strains):** Drinking water: 4 log_10_ reduction until day 10. Then gradual increase back to baseline on day 20. Oral gavage: Maximum reduction was on day 7, at 3.45 log_10_ Vegetable capsules: Maximum reduction 4.62 log_10_ on day 6. Then gradual increase back to baseline on day 20	Phage shedding peaked around 10^5^ PFU between days 4-12 for all administration routes. Then it gradually declined until day 20 (0 PFU), following a reverse course than E. coli CFU counts.	N/A
[81]	*E. coli*	Mouse GI tract infection	3 dosing regimens: Single administration of 10^8^ PFU/mouse. Single administration of 10^10^ PFU/mouse Daily administration of 10^10^ PFU/mouse, orally	Only daily administration of phage reduced bacterial burden in GI tract (1-2 log_10_)	Daily administration pf phage kept phage concentration at 10^5^ PFU/ml until day 5. Single administration of 10^10^ kept phage concentration at 10^3^PFU/ml. No phage was detected after day 3 with single dose of 10^8^ PFU/ml	N/A

Abbreviations: IP: intraperitoneal; PFU: plaque forming unit; N/A: not applicable; IM: intramuscular; IV: intravenous; CFU: colony forming unit; GI: gastrointestinal; UTI: urinary tract infection.

The route of administration in these studies depends on the type of infection; eg, orally or intraperitoneally administered bacteriophages are used to treat GI tract infections and urinary tract infections (UTIs) [42, 43, 60, 63]. Eye drops are used for bacterial keratitis [69] and intramuscular administration is used for soft tissue infections [70–72]. Phage doses range from 10^7^ – 10^11^ PFU/animal. Notably, a study by Bull et al [70] administered a dose as low as 100 PFU/animal intramuscularly in an *E. coli* thigh infection model, to examine the effect of the K1 antigen-specificity of the phage in the therapeutic outcome, showing that phages requiring the K1 capsule (K1-dependent phages) rescue mice even when administered at doses 10^6^-fold lower than the K1-independent ones.

The result of most treatments was either the increase in survival of treated animals, or the decrease in CFU compared with the non-treated controls. The exception to this observation is the study by Reynaud et al [73], studying a rabbit ileal loop model of *E. coli* infection, in which phage administration only managed to postpone the death of the animals for 3 days, but did not significantly increase the survival rate compared with the untreated group. Moreover, Jamalludeen et al [74] demonstrated a reduced duration and severity of *E. coli* diarrhea and lower CFU counts in a porcine model, compared with the untreated group.

With regards to phage kinetics, phages which encountered bacteria remained in tissues longer than those not encountering bacteria. Notably, no difference in phage clearance was observed in the study by Bull et al [70] between K1-dependent and K1-independent phage.

Three of the studies examined the histology or cytokine levels of animals in treated and untreated groups. In an *E. coli* peritonitis mouse model by Matsuda et al [76], IL-6 was significantly reduced in the phage-treated groups, whereas the use of a Lysis-Deficient (LyD) phage led to lower levels of TNF-α compared with mice with peritoneal infection treated with the wild type (WT) phage for *E. coli.* This was achieved due to the use of a phage mutant, which does not lyse bacterial cells, and therefore does not result in the release of endotoxin and consequent elevation of pro-inflammatory cytokine levels. It does, however, kill the bacteria, by cleaving their DNA with phage-induced enzymes, as well as using bacterial metabolism for phage replication. Rastogi et al [43], in their rat model for phage treatment of GI infection with *E. coli*, reported that phage-treated groups showed no damage or lesions in the GI tract, in comparison with untreated controls, which showed lymphomononuclear infiltration and edema. Hung et al [77], in their study for treatment of liver abscesses attributed to *K. pneumoniae,* measured circulating cytokines, as well as hepatic enzymes, and reported lower levels of TNF-α, IFN-γ, MCP-1, IL-10, and IL-6, as well as aspartate aminotransferase (AST) and alanine aminotransferase (ALT) in phage-treated mice. Finally, in a murine model of *P. aeruginosa* keratitis, established by Fukuda et al [75], phage-treated mice had normal corneas, compared with a destroyed stromal structure and severe neutrophil infiltration of the corneas in untreated mice.

### Human clinical trials and case reports

Following the promising results of phage treatment from the *in vitro* and preclinical *in vivo* data from animal models, bacteriophages have been tested in the clinical setting, particularly in patients whose infections do not respond to antibiotics. There have been several case reports of successful bacteriophage therapy in patients with different infections, and safety trials for phage targeting Gram-negative bacteria [82–84]. These reports focus on patient outcome and did not typically examine the kinetics of phage therapy as the urgency under which the bacteriophages were administered to patients precluded appropriate sampling of tissue or serum for bacteriophage titer. Likewise, histologic analysis and cytokine measurements were performed in only 2 of the 15 included reports.

Fifteen case reports and clinical trials for bacteriophage therapy were reviewed and are summarized in Table 5. Three studies involved pneumonia caused by *P. aeruginosa* [85–87], two reported the treatment of burn wound infections attributed to the same pathogen [82, 88], two dealt with bone infections, a left tibial lesion and a leg ulcer, both of which were polymicrobial infections. One case involved bacteriophage for treatment of necrotizing pancreatitis caused by *A. baumannii* [89], one applied phage for UTIs [90], another for treatment of acute diarrhea [91], 1 for treatment of *P. aeruginosa* septicemia [92], and one for treatment of hospital-acquired infections in an intensive care unit [93]. Another study reported the use of therapeutic bacteriophage against otitis caused by *P. aeruginosa* [94], and three studies were safety trials in healthy volunteers [83, 84, 51].

**Table 5.** Human Studies

Reference	Pathogen	Animal Model	Dose & Route of Administration	Outcome	Phage Kinetics	Histology & Cytokines
[82]	*P. aeruginosa*	Burn wound infection	10^2^ PFU, topically, once daily for 7 days (compared with the anticipated 10^6^, phage titers were lowered after long storage)	Phage-treated group had a longer time before 2 quadrant reduction in CFU was achieved (144h), compared with standard of care (47h). Furthermore, only 50% of phage group patients sustained that reduction, compared with 85% of standard of care 23% of phage-group had adverse effects, compared with 54% of standard of care	N/A	N/A
[85]	*P. aeruginosa*	Pneumonia in CF patient	4 × 10^9^ PFU, IV, every 6 hours, for 8 weeks	The patient had no fever on day 7 and oxygen administration was reduced on day 8. The patient became ambulatory, was discharged from the hospital, and did not develop pneumonia for the 100-day follow up period; 9 months later she received a lung transplant	N/A	N/A
[83]	*E. coli*	Safety trial - Healthy adult volunteers	3 × 10^9^ PFU (high dose) or 3 × 10^7^ PFU (lower dose), orally	No adverse effects in self-report, clinical evaluation and liver, kidney, and hematological examination	64% of higher dose volunteers' feces had phage at or below 3 × 10^7^ PFU/mL only 30% of lower dose had phage (median phage concentration = 10^2^ PFU)	N/A
[84]	*E. coli*	Safety trial - Healthy adult volunteers	10^5^ or 10^3^ PFU/volunteer, orally	No adverse effects were reported, except for a volunteer with a sore throat. Liver enzymes were normal in all volunteers	10^5^ PFU: 100% of volunteers had phage in their feces on day 3 (approximately 10^4^ PFU/mL), which was retained until day 7 10^3^ PFU: 50% prevalence in feces on days 2 and 3, phage was completely cleared by day 5	N/A
[86]	*P. aeruginosa*	Pneumonia	1st patient: 10^9^ PFU, IV, every 6h nebulized every 12h, with concomitant antibiotics 2nd patient: 10^9^ PFU, IV, every 12h	1st patient: Improved within 29 days and had become ambulant by day 16. He presented back with pneumonia on day 46 (mucoid MDR *P. aeruginosa*) and re-started treatment. He was discharged from the hospital on day 150 after he improved and there was no active pneumonia for 3 months 2nd patient: Improved within 90 days, and she was discharged from the hospital. She came back with another infection that was non-MDR *P. aeruginosa* related. For the following 7 months, she was free of infection	N/A	N/A
[87]	*P. aeruginosa*	VABP	10^9^ PFU, IV, and 4 × 10^9^ PFU, nebulized, every 12 hours, with concomitant use of antibiotics	Antibiotics alone did not improve the condition of the patient, but 3 days after phage initiation the patient improved dramatically, her %SaO2 increased and she was removed from intubation	N/A	N/A
[88]	*P. aeruginosa*	Safety trial - Burn wound infection	Single dose of 10^7^ PFU, topically on half the wound (wound was divided into 2 sections: 1 received phage and the other standard treatment with amikacin, ceftazidime and meropenem)	CFU counts were not significantly reduced after the use of phage	N/A	N/A
[89]	*A. baumannii*	Necrotizing pancreatitis in diabetic patient	4 × 10^9^ PFU, initially through percutaneous catheters, after non-responsiveness, phage IV was also administered for a total of 18 weeks	The patient awoke from coma and became conversant. The patient's renal function (with an initial serum creatinine of 3.68 mg/dL) improved, and his general condition also improved gradually. He was discharged from the hospital on day 245 and resumed his normal life.	5 min after administration, plasma levels of phage were 10^4^ PFU/mL, and phage was almost completely eliminated from plasma within 60 min. After 6 hours, no phage was detected.	N/A
[90]	*P. aeruginosa* and *E. coli*	UTI	10^7^ – 10^9^ PFU, through transurethral catheter, every 12h	2-3 log_10_ reduction in CFU or complete eradication of the pathogen in some patients	N/A	N/A
[91]	*E. coli*	Safety trial - Acute diarrhea - children	Orally, 2 different cocktails, one of each group: Russian Microgen phage cocktail (M) and T4-like phage cocktail (T)	No significant differences in duration of the diarrhea or clinical score of the patients or the quantitative factors (eg, diarrhea frequency, stool weight etc) were observed between the groups	M group: highest phage titer of all groups and 100% phage-positive stool for 3 days. T group: Higher than placebo, as well as in the number of days with phage-positive stool rate	Phage recipients had normal liver, kidney, and blood test results and did not have a positive Jarisch-Herxheimer reaction
[92]	*P. aeruginosa*	Septicemia	5 × 10^7^ PFU, IV, every 6h and topically on wounds every 8h	Blood cultures became negative, the fever dropped, and the patient's kidney injury subsided	N/A	N/A
[93]	*P. aeruginosa* *K. pneumoniae* *A. baumannii*	Hospital-acquired infections	5 × 10^8^ PFU, orally, phage cocktail with 2 phages for each of the pathogens	Before treatment, 79% of samples from patients' endotracheal aspirate, blood, and urine were infected. After treatment, this rate dropped to 29%	Phage was isolated from endotracheal aspirate, blood, feces, and urine 5 days after administration, showing that they cross the gastrointestinal epithelial barrier	N/A
[94]	*P. aeruginosa*	Otitis	1.2 × 10^4^ PFU, topically	Visual Analog Scores were improved in patients of the treatment group. The phage-treated group showed improvement in inflammation, erythema, odor, and ulceration/granula-tion/polyps, compared to placebo group. CFU was reduced from 80-100% in phage-treated patients, compared with non-treated ones	Mean PFU recovered from patients was 1.27 × 10^8^, suggesting an amplification of approx. 200 times compared with the initial dose	N/A
[51]	*E. coli*	Safety trial - Healthy adult volunteers and healthy children	7 × 10^6^ or 7 × 10^5^ PFU, orally	No adverse effects in most of the patients. Abnormal eosinophil counts and liver function values were observed, but were present before the start of the treatment. One patient each reported a toothache, upset stomach, and ear infection. In general, no serious adverse effects were reported	N/A	N/A
[95]	*P. aeruginosa* and *E. coli*	Leg ulcers – Safety trial	Topically (dose is not mentioned)	No difference in the healing rate between the 2 groups. They both had similar percentages of patients healed by week 12 and by week 24	N/A	N/A
[96]	*A. baumannii* and *K. pneumoniae*	Left tibial osteomyelitis	5 × 10^7^ PFU, IV over 35 minutes, for 5 days and then continued for an additional 6 days after Ab cultures were positive mero-penem (2 grams tid), IV and colistin (4.5 × 10^6^ units/bid), IV, were also administered	Wound recovery started within few days of treatment and the wound eventually healed completely and patient's pain disappeared; 8 months post-treatment, the patient did not show any re-infection with either organism	N/A	N/A

Abbreviations: PFU: plaque forming unit; N/A: not applicable; IV: intravenous; CFU: colony forming unit; CF: cystic fibrosis; MDR: multidrug resistance; %SaO2: percent saturation of oxygen

All studies involving therapeutic use of bacteriophage, administered the cocktail intravenously at various dosing intervals of 6, 8, or 12 hours, or through the route that was consistent with the site of infection. Thus, wound infections were treated with topical administration of bacteriophage, in UTIs through trans-urethral catheters, and in pneumonias via the intravenous or nebulized routes. In all therapeutic applications of bacteriophage, the dose ranged in the order of 10^7^ – 10^9^ PFU, with the exception of the study by Jault et al [82], where the administered dose was 10^2^ PFU, due to a marked reduction in the phage titer after long storage.

In all but 1 reported case of therapeutic application of phage, the patients recovered from a critical condition without re-infection for follow-up periods that were as long as 9 months. Only 2 of the therapeutic application studies examined the kinetics of phage. In the study by Schooley et al [89], phage concentration in plasma was reduced to less than half the initial dose within 5 minutes and was almost completely eliminated within 60 minutes. After 6 hours, no phage could be found in the plasma. Aleshkin et al [93] were able to isolate phage from the endotracheal aspirate, blood, feces, and urine for up to 5 days after administration through an orogastric tube. These observations demonstrate that bacteriophage can translocate across the gastrointestinal epithelium. None of these studies examined the histological and immunological aspects of phage therapy in humans.

Both healthy volunteers and sick patients participated in safety trials of bacteriophage therapy. The doses used in those cases had a wider range, between 10^3^ and 10^9^ PFU, and were administered either orally or topically, as in the case of burn wound infections [88] or leg ulcers [95]. These studies mainly examined the safety of phage administration. Evaluation of the safety of bacteriophage was determined by clinical assessment, as well as by biochemical parameters, including hepatic and renal function, and hematological parameters. Phage therapy was well tolerated with few mild self-limiting adverse events reported [51, 84].

During phage therapy of a patient with disseminated resistant *A. baumannii* infection, emergence of resistance occurred to the first bacteriophage cocktail. In order to overcome resistance, 2 consecutive phage cocktails were administered during the course of the patient's extended treatment [89]. Since emergence of resistance of infecting bacteria to therapeutic phages is a potential limitation to phage therapy, rational strategies are needed to anticipate and prevent these events.

The 3 studies which reported phage kinetic data [83, 84, 91] demonstrated that after oral or IV administration, phage persisted in all patients' feces for 3 days and for as much as 7 days in many cases. In terms of immune response evaluation, only Sarker et al [91] reported that the children in their study did not have a positive Jarisch-Herxheimer-like reaction, which would be indicative of endotoxin release after the lysis of bacterial cells. No other studies in humans have examined the histology or cytokine immune response of the patients.

### Safety

Current studies thus far have demonstrated that intravenously administered phage therapies for Gram-negative bacillary infections, have minimal adverse effects, thus avoiding possible toxicity to seriously ill patients with multidrug resistant Gram-negative bacterial infections, something that could complicate treatment. Among studies that examined safety of phage application, the following adverse events were reported: A transient hypertension in a patient with *A. baumannii* infection, which did not require vasopressors, and an increase in blood pressure in a different patient with an *A. baumannii* infection, which lasted for approximately a week but was resolved after temporary discontinuation of the phage administration. In a patient with a *P. aeruginosa* infection, a 38.5°C fever was observed, as well as chills, on the third day of phage therapy. In the most serious side effect observed so far, concerning a 2-year-old child with a *P. aeruginosa* infection, phage therapy was withheld due to anaphylaxis-related decompensation, which was attributed to progressive heart failure. Finally, 1 patient reported mild headaches, and another patient reported transient ear pain during phage therapy. In most cases, those side effects are attributed to the endolysin release after killing of the bacteria by the phage. These side effects may be reduced or completely minimized when using a lysis-deficient phage, as was the case in the study by Matsuda et al. Independent of the route of administration, phages are a safe therapeutic intervention [83, 84]. In the studies that monitored potential side effects with laboratory tests, such as in a randomized trial for oral phage therapy of acute diarrhea in children [91], phage recipients had normal liver, kidney, and blood test results and did not have a positive Jarisch-Herxheimer-like reaction.

As the result of being highly targeted to a specific host bacterial pathogen, bacteriophage therapy is less likely than conventional small molecule antimicrobial agents to significantly disturb the normal gastrointestinal microbiota. By comparison, broad spectrum antimicrobial agents that are currently used to treat multidrug resistant Gram-negative bacterial infections, inevitably lead to disruption of the gastrointestinal microbiota and potentially lead to antibiotic-associated diarrhea, including *Clostridium difficile-*associated diarrhea.

### Pharmacokinetics and pharmacodynamics

In terms of the pharmacological aspects of bacteriophage therapy, phages and conventional antibiotics share some common properties that govern their application. Both bacteriophages and antimicrobials display either concentration-dependent or time-dependent killing. In certain infections, depending on the organism and type of infection, phage treatment may be concentration-dependent and similar to the C_max_/MIC ratio in conventional antibiotics, such as amino-glycosides or fluoroquinolones. In this case, the multiplicity of infection or MOI (ie, the ratio of phage particles divided by the pathogen load at the time of infection) may play an important role in the outcome of the treatment. This is similar to the effect of the peak plasma concentration (C_max_) of an antibiotic, divided by its minimal inhibitory concentration (MIC), as observed in concentration-dependent antibiotics. Contrary to that, time-dependent activity (defined as the time spent above the MIC in the case of conventional antibiotics) means that dosing intervals should be set in such a way that phage concentration is retained above the necessary MOI long enough to achieve killing of the organism (Figure 2). Deriving this is complicated by ongoing phage replication in the target pathogen during treatment, and it is likely that this replication rate varies by compartment and even by microenvironment. Dose optimization may be conducted by serial quantification of bacteriophages in blood and tissue, as demonstrated. Alternatively, a more common approach for dose optimization of bacteriophage is conducted *in vivo* through empirical evaluation of dosages and dosing intervals.

**Figure 2A.**
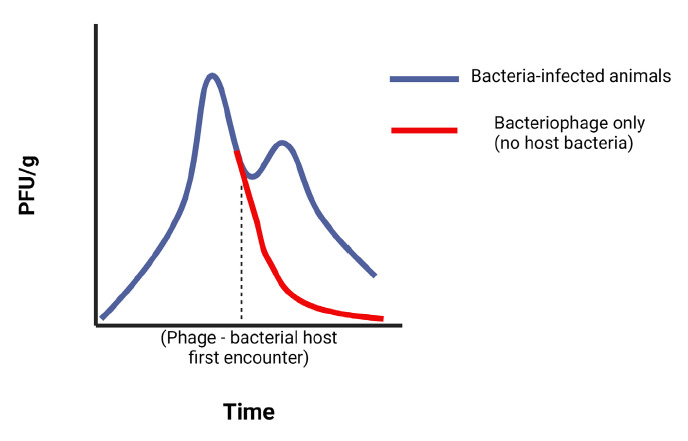
**Conceptual model of the effect of bacterial infection on phage (Φ) kinetics in blood and tissues.** When phage encounter their bacterial hosts (bh) in infected animals, they replicate, and therefore require a longer time to be cleared from the system, in comparison to that of uninfected animals, where there are no bacteria that phage can infect. This self-replication property extends their therapeutic effect, as it leads to higher titers in tissues and blood, potentially leading to a reduced need for many doses, when compared with conventional antibiotics.

**Figure 2B.**
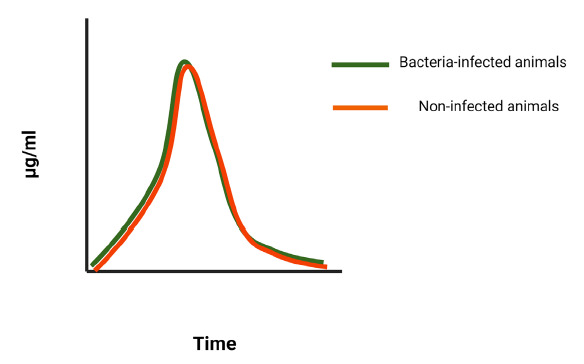
**Conceptual model of the kinetics of conventional antibiotics in blood and tissues.** In comparison to bacteriophages, conventional antibiotics cannot replicate and are cleared from the system within the same time, regardless of whether or not a bacterial infection is present in animals.

**Figure 3.**
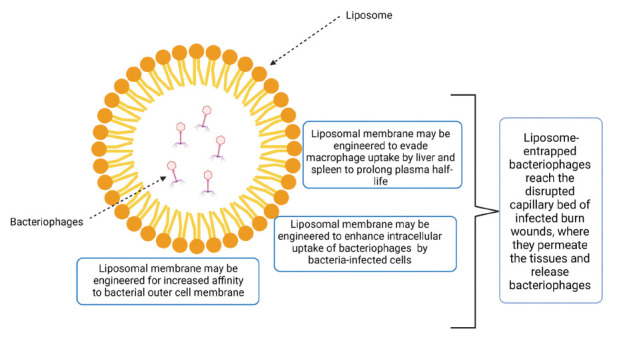
**Possible mechanisms of enhanced antimicrobial activity of liposome-entrapped bacteriophages (LEBs).** The chemical composition and structure of liposomes of LEBs may be engineered for polarity, size, and lipophilicity to evade macrophage uptake by liver and spleen to prolong the plasma half-life of bacteriophages, to enhance intracellular uptake of LEBs by bacteria-infected cells, and to increase affinity to bacterial outer cell membranes. Moreover, LEBs reach the disrupted capillary bed of infected burn wounds where they permeate the tissues and release bacteriophages.

Another similarity between bacteriophages and antibiotics that this review highlights, is the need for pharmaceutical formulations which will assure that the antimicrobial will be delivered to the infected target. For example, providing an enteric film coating, which will protect an antibiotic from degradation in the stomach and help release it in the small intestine, can also be applied to bacteriophage [78, 95]. Likewise, coating of the phage with liposomes (Figure 3), as mentioned in several studies reported herein, can release the phage in a more targeted way, reducing the phage concentrations needed for activity. Such is the case in the studies by Singla et al [47] and Chadha et al [57], which showed that liposome entrapment can improve therapeutic outcomes even when the phage concentration and MOI are much lower than those used in non-liposome entrapped therapies.

In many of the studies mentioned above, phage kinetics with regard to *bacterial host encountering* was evaluated. More specifically, these studies demonstrated that when phage encountered host bacteria, they replicated and therefore remained in circulation and in tissues longer than if there was no bacterial host. To optimize the phage-host interaction would require study of the optimal initial dose to combat a specific bacterial CFU, while also investigating the increase of MOI as bacteriophage replicate in host bacterium they encounter. More extensive studies that address these pharmacokinetic properties of bacteriophage and multidrug resistant bacteria are necessary to make further advances in the field of phage therapeutics.

Finally, another distinctive property of phage therapy may be the manipulation of phage dose based on host membrane structure. As shown by Bull et al [70], specificity of the phage to a bacterial surface feature that was also required for virulence greatly enhanced the outcome of treatment. This finding suggests that low PFU doses, and thus less phage product, may be as effective as high PFU doses in well-tailored phage-therapy treatments.

### Current challenges, usages, and directions for phage therapy

While animal studies and individual case reports have indicated that phage therapy can be effective for the treatment of bacterial infections, the path ahead presents multiple challenges. Commercial development of therapeutic phages is an ongoing process: phage products are already available for food safety applications [72], for the control of plant pathogens, for use in aquaculture, and as nutritional supplements. These areas have been more attractive for initial commercialization as the regulatory requirements for marketing in these sectors are generally much simpler than for medicines. As of this writing, there are 19 clinical trials registered on clinicaltrials.gov for the evaluation of phage-based therapeutics to treat a variety of infection types; 7 of these clinical trials are recruiting patients.

The high specificity of phages, seen as an advantage for avoiding collateral damage to non-pathogenic flora, is also a challenge for the development of phages as broadly applicable therapeutics. Because a given phage will only infect a subset of strains within a bacterial species, multiple phages will be needed to adequately cover the most clinically relevant strains of a pathogen. Given the propensity for bacterial resistance to individual bacteriophages, optimized clinical application of phages against MDR Gram-negative bacilli will likely require the use of phage mixtures, or “cocktails”. Cocktail phages may be chosen based on their host range across multiple pathogen strains, their ability to forestall the emergence of resistance, their favorable PK/PD characteristics, and their ability to minimize the probability of emergence of resistance within bacterial populations causing infection [43]. The desired properties may be obtained by identifying and combining naturally-occurring phages, or by engineering phages to possess the desired characteristics [48]. Many aspects of basic phage biology remain to be discovered to fully exploit their therapeutic potential, but phage engineering is also attractive because it provides much stronger intellectual property (IP) protection for the final product, a significant concern in commercial drug development.

Treatment of Gram-negative bacterial infections with phage may result in the rapid release of lipopolysaccharide with subsequent pro-inflammatory and hemodynamic deterioration in the infected host. This deleterious effect may be circumvented by lysis-deficient (LyD) phage. Further development of such phage cocktails may be a safer alternative to the use of lytic phages.

Two broad models of phage development have been proposed [97]. The first model resembles a more traditional, fixed-composition product designed to treat a specific pathogen or condition. Such a product would have a clearly defined identity and would move through a regulatory approval process that resembles that used for other small-molecule drugs or biologicals. This approach has the advantages of clearer pathways for both regulatory approval and IP protection. The disadvantage of this approach is the static nature of the product, which may not be able to treat all pathogen strains circulating in a patient population and which cannot adapt to the emergence of new strains that are not susceptible to the phages in the product. This disadvantage may be allayed in part by the implementation of a more flexible regulatory framework that would allow for periodic updating or *versioning* of the product to adapt to current needs without the need for completely new sets of preclinical and clinical studies. The second proposed model is a more flexible, personalized medicine approach in which a mixture of therapeutic phages is formulated on-demand based on the phage sensitivity of an individual patient's pathogen isolate. This model has the advantages of adaptability to individual patient needs and is thus less likely to be hampered by individual strain variability, but details on how to scale this approach to a level that would make it broadly available are yet to be determined. Additionally, the regulatory pathway for this approach is much less defined, as there is no single fixed-definition molecular entity to be approved. The magistral preparation approach used in some European Union jurisdictions may be compatible with phage personalized medicine approaches [44], but the approval process for this implementation in other jurisdictions remains to be clarified. Magistral preparation refers to the formulation of a medicinal product by a pharmacy that fills a prescription for a specific patient and not requiring marketing or manufacturing licensing or authorization.

Another approach of harnessing the potential power of phage therapy is to administer modified lysins for treatment of life-threatening infections. Following successful preclinical investigations, a phase 2 clinical trial of lysin plus standard antibacterial therapy was found to be more active than standard antibacterial therapy alone in treatment of MRSA bacteremia and right-sided endocarditis [98]. These results led to a phase 3 clinical trial, which unfortunately was closed for lack of feasibility. Further preclinical studies are warranted to optimize the activity of lysin in MRSA infections as a guide to future clinical trials. Current and future preclinical studies are underway to investigate the antimicrobial activity of lysins and amurins for treatment of infections caused by *P. aeruginosa* and *A. baumannii* [49].

Finally, economic issues that affect anti-infective development will also affect phage therapy. The stand-out application of phage therapy is the treatment of multidrug-resistant or pan-resistant organisms, which are not responsive to treatment by conventional antibiotics. While the threat of antimicrobial resistant (AMR) bacteria continues to grow, these infections represent only a portion of all cases, and thus the current market for novel therapeutics against these infections is not as large as the market for antibacterial agents as a whole. That antibacterial development has been seen as economically unattractive poses a challenge for conventional commercial investment [46]. However, as the frequency of AMR infections continues to rise, it would seem prudent to invest in new antibacterial strategies in order that they can be in place for future generations.

## CONCLUSIONS AND PERSPECTIVE

The global emergence of multidrug-resistant Gram-negative bacterial infections threatens the limited antimicrobial armamentarium. Bacteriophage therapy offers a compelling adjunct to development of new antimicrobial agents to meet this global challenge. Depending upon the type of infection, the level of resistance by the target bacteria, and efficacy of available antibiotics, clinical trials of phage therapy may be designed as adjunctive therapy or monotherapy. Animal model systems can be used as powerful tools for the development of new bacteriophage therapies, as well as for designing and lowering the risk of clinical trials. Bacteriophage therapy for human use has currently been limited to a small number of individual cases, but these treatments have demonstrated the proof-of-concept that phages can be used to combat life-threatening antimicrobial resistant bacterial infections. Going forward, a focus on pre-clinical pharmacokinetics and pharmacodynamic optimization of bacteriophage therapy against multidrug-resistant bacterial infections, using quantifiable endpoints of residual tissue bacterial burden, survival, and pro-inflammatory cytokine profiles, will be critical in obtaining a better understanding of bacteriophage efficacy in a clinical setting. Additionally, the development of highly predictive animal model systems, well-designed clinical trials, and reliable sources of produced bacteriophage will be essential in further improvement of this promising technology in combatting multidrug-resistant Gram-negative infections.
